# The Association between Mental Motor Imagery and Real Movement in Stroke

**DOI:** 10.3390/healthcare9111568

**Published:** 2021-11-17

**Authors:** Ana Poveda-García, Carmen Moret-Tatay, Miguel Gómez-Martínez

**Affiliations:** 1 Escuela de Doctorado, Universidad Católica de Valencia San Vicente Mártir, San Agustín 3, Esc. A, Entresuelo 1, 46002 València, Spain; 2 Facultad de Psicología, Universidad Católica de Valencia San Vicente Mártir, Avenida de la Ilustración, Burjassot, 46100 Valencia, Spain; mariacarmen.moret@ucv.es; 3 Dipartimento di Neuroscienze Salute Mentale e Organi di Senso, La Sapienza Università di Roma, 00185 Rome, Italy; 4 Departamento de Terapia Ocupacional, Centro Superior de Estudios Universitarios La Salle, 28023 Madrid, Spain; miguel.gomez@lasallecampus.es; 5 Occupational Thinks Research Group, Centro Superior de Estudios Universitarios La Salle, 28023 Madrid, Spain

**Keywords:** mental motor imagery, neurorehabilitation, stroke

## Abstract

Background: Stroke is the main cause of disability in adults; the most common and long-term sequela is upper-limb hemiparesis. Many studies support the idea that mental motor imagery, which is related to the visualization of movement patterns, activates the same areas of the cortex as if the movement occurred. Objectives: This study aims to examine the capacity to elaborate mental motor images, as well as its relationship to loss of movement in the upper limbs after a stroke. Method: An observational study, in a sample of 39 adults who suffered a stroke, was carried out. The upper limb movement and functionality, cognitive disorders, the ability to visualize mental images, and activities of daily living were examined. Results: The results depicted a statistically significant correlation between the ability to visualize upper limb mental motor images with movement, functionality, and strength. In addition, a correlation between visual–spatial skills and mental visualization of motor ability and upper limb movement was found. Conclusions: These results suggest that the rehabilitation approach focused on the improvement of mental motor imagery could be of interest for the upper limb rehabilitation of movement and functionality.

## 1. Introduction

A stroke occurs when the blood supply to part of the brain is interrupted or reduced, preventing brain tissue from receiving oxygen and nutrients. Brain cells begin to die in minutes. There are two main causes of stroke: ischemic, which occurs when a vessel supplying blood to the brain is obstructed, and hemorrhagic, in which a weakened blood vessel supplying the brain bursts. The consequence of stroke in both cases is a neurological deficit derived from the fact that a part or area of the brain stops working properly. The neurological deficit is one of the main causes of disability worldwide. [1].

There are over 13.7 million new strokes of all types each year worldwide. Every year, over 116 million years of healthy life are lost due to stroke [2]. The predominant long-term disability, which usually features the worst prognosis, is upper limb (UL) movement [3,4]. Approximately only one-third of people affected by stroke achieve fully functional UL recovery [3,5].

Thus far, a wide range of strategies and devices have been developed for the purpose of promoting upper limb motor recovery after stroke by taking advantage of the brain’s ability to reorganize its neural networks after injury. The functional organization of the motor system is modified by use, and it has been suggested that use-dependent plasticity may play a major role in the recovery of function after stroke [6,7,8].

This approach includes neuromodulation techniques, such as transcranial magnetic stimulation or transcranial direct current stimulation, or sensory transformation techniques, such as mental practice/mental imagery or mirror therapy, which enhance use-dependent plasticity. More precisely, it should be noted that motor imagery (MI) is the mental representation of an action without a physical movement or muscular activation.

Research supports the idea that motor imagery should, to some extent at least, involve the same neuronal substrate as an executed movement [9]. Indeed, several studies reported that greater activation is achieved in the supplementary motor area (SMA), the premotor cortex (PMC), and the primary motor cortex (M1) in subjects during both executed and imagined movement [10,11,12,13,14]. Some brain areas that are activated during motor imagery belong to the neural network known to be involved in the early stage of motor control (i.e., motor programming) [10].

This finding has opened many interesting lines of research, which can be described as follows: (i) knowledge of MI for the application of therapy [15,16,17,18,19]; (ii) evidence regarding the neurophysiological bases of MI [20,21,22,23]; (iii) knowledge regarding the relationship between MI and physical movements [24,25]; (iv) analysis and validation of assessment tools to measure the ability to visualize movements [26,27]. Thus, this research aimed to examine the relationship between MI and real UL movements in individuals affected by stroke. Considering that the visualization of a movement can activate neurons in the same motor areas, the purpose of this study was to address the following research question: what happens if we cannot create the mental MI, as in the case of stroke? Would the ability to move be affected? In other words, this work aimed to deepen the knowledge about the relationship between mental imagery and motor deficits after stroke.

Many studies support the idea of functional equivalence between motor images and motor execution [28,29,30]. Most of us can imagine moving our fingers typing on a computer, but apparently, we cannot imagine typing faster than we can actually move our fingers [31,32]. A study that analyses the functional equivalence between images and action concludes that if an action cannot be physically performed, it cannot be imagined with a high functional equivalence [33]. Most of these studies have been carried out with healthy participants, analyzing functional equivalence by checking the temporal coupling between motor visualization and their subsequent motor performance of a task [32,34,35]. The innovative approach of the current research is focused on investigating the lack of ability to visualize motor patterns, and how this is related to an actual loss of UL movement and functionality. To this end, it is expected that people who suffered a stroke and are not capable of visualizing movement experience a greater UL handicap, with poor fine motor skills, muscle weakness or spasticity, and loss of functionality and independence.

## 2. Materials and Methods

### 2.1. Ethics

This research was conducted in Los Madroños Hospital, a leading medical center based in Madrid (Spain), specializing in neurological pathologies and their neurorehabilitation. The research was approved by the Research Ethics Committee of the Higher Center for University Studies La Salle Madrid.

The participants received an informative form on the goals of the current research. Informed consent was compulsory. The participants were volunteers and were not coerced; they were free to leave at any moment.

### 2.2. Study Design

This study aimed to analyze and correlate UL movement scales, cognitive instruments, and evaluations of the capability to visualize MI. Additionally, activities of daily living (ADL) independence measures, along with information related to demographic characteristics such as months since the injury, were also considered in this research. A direct relationship between MI and movement was expected, since, according to the previous literature, patients with this profile are considered to have higher difficulty in performing movements if they do not visualize them.

An observational approach was chosen. Lastly, participants were divided into two groups of early post-stroke patients (up to 6 months after stroke) and late post-stroke phase (e.g., over 6 months) to examine motor imagery over time.

### 2.3. Data Analysis

Data analysis was conducted using statistical software, IBM SPSS Statistics version 20 (IBM, Armonk, NY, USA). A Bivariable analysis and Pearson correlation coefficient were employed. In addition, the participants were divided into two groups considering their ability to perform MI. Data normality was examined, and depending on their results, the non-parametric Mann–Whitney U or the parametric *t*-test were used to determine statistical significance. Lastly, a 2 (MI groups) x2 (time after injury groups) ANOVA was carried out on the scores of Mental Evoking Images, Movements and Activities Questionnaire. The statistical significance level was set at *p* < 0.05.

### 2.4. Participants

Inclusion criteria:

- People over 18 years old;
- Participants who have suffered an ischemic or hemorrhagic stroke, with no time limit since the injury;
- Medically stable participants who can attend therapy;
- Participants who are able to give informed consent.

Exclusion criteria:

- Participants with previous pathology in their upper extremity, traumatic, neurological, or any other type of pathology that may affect the results of the assessment;
- Participants who have reported some neurological alteration before the stroke;
- Severe aphasia, memory disorders, attention disorders, visual and communication disorders, or other neural symptoms, which may interfere with this study.

A final sample of 39 adults volunteered to participate in the study (11 females and 28 males). Participants’ age ranged from 49 to 84 years old (*M*_Age_ = 66.15; SD = 9.88). All the participants had suffered a stroke (19 ischemic and 20 hemorrhagic) and had different hemiparesis levels and showed different half-body affected (22 participants with left half-body affected and 17 with right half-body); the neuroimaging results were very varied, and in some cases, the diagnosis was not very precise. The main areas of involvement were the left and right middle cerebral artery (23%), nucleus basalis (12%), and others (64%). Time since injury ranged from 1 month to 4 years since the stroke (*M*_Months_ = 7.87 SD = 9.20). None of them had a previous pathology in their upper extremity (traumatic, neurological, or any other type that may affect the results of the assessment). It should be also noted that all the participants were medically stable, so they could attend therapy.

### 2.5. Measures

For data collection, the participants were examined using several assessment methods and tests, which were conducted at the Los Madroños Hospital. The employed assessment tools are described in the following subsections.

#### 2.5.1. Upper Extremity Evaluations

A total of five instruments to evaluate the most affected UL were used: (1) Fugl–Meyer assessment for the upper extremity (FMA-UE) [36], which is adapted and validated for the Spanish population. This is a stroke-specific performance-based impairment index that has been extensively tested and found to have excellent properties. This is a quantitative examination instrument with a maximum score of 66 points. It is designed to assess motor functioning, sensation, and joint functioning in subjects with post-stroke hemiplegia. It is applied for both clinical and research proposes to determine disease severity, describe motor recovery, and plan and assess treatment; (2) ABILHAND scale [37], which is also translated into Spanish. This is an appropriate outcome measure for assessing upper extremity performance in daily activities in subjects with stroke. It is an interview-based assessment in which the patient is asked to estimate the ease or difficulty of performing a list of activities when carried out without assistance. This can involve any strategy used to carry out the activity; it is a self-report and not a physical demonstration of the activity; (3) nine-hole peg test (9-HPT) [38], which is translated into Spanish. This is a standardized, quantitative assessment used to measure finger dexterity that requires participants to repeatedly place and then remove nine pegs into nine holes, one at a time, as quickly as possible; (4) the Jebsen hand function test (JHFT) [39], which is a translated tool into Spanish and a standardized evaluative measure of functional hand motor skills. JHFT consists of 7 items that measure fine motor skills, weighted functional tasks, and non-weighted functional tasks; (5) dynamometry test [40], for which a Deyard Tech digital dynamometer (range 5–90 kg) was used in order to assess the ULs grip strength. During the dynamometry test, the position of the participant was seated with his back resting on the backrest and their feet on the floor, the arm resting on a table in front of them, with the elbow flexed at 90° and the wrist in a neutral position. The assessment consisted of 2 tests where the subject pressed a manual dynamometer, once with each hand, and the best strength result of the 2 tests performed with each hand was taken.

#### 2.5.2. Motor Imagery Ability

In mental health practice, different tools are usually used to evaluate the capability to perform MI such as the Imagery Use Questionnaire (IUQ) [41], Movement Imagery Questionnaire (MIQ) [42], Movement Imagery Questionnaire-Revised (MIQ-R) [43], Vividness of Movement Imagery Questionnaire (VMIQ) [44], and Kinesthetic and Visual Imagery Questionnaire (KVIQ) [27].

For the proposes of the current research, the Mental Evoking Images, Movements and Activities Questionnaire (CEMIMA) was used due to several reasons: First, CEMIMA is a psychometrically robust instrument that can be used to measure the ability to create visual and kinesthetic mental images of the UL. Second, CEMIMA can evaluate the capability to visualize the isolated UL movement. Third, CEMIMA allows the examiner to collect qualitative information about the MI visualization process, including the difficulties that the participants may have, such as interferences or disruptions during the MI visualization.

CEMIMA scale was developed and validated in the Spanish language. The process of data collection was divided into 5 different steps: steps 1 and 2 encompassed data collection on mental visualization of one of the hands; step 3 included data collection on movement visualization of selected hand; in steps 4 and 5, data on mental visualization of the selected hand during an activity (e.g., moving a teaspoon or throwing and catching a ball) were collected. The questionnaire had positive/negative scores. Positive scores were based on a Likert scale from 0 to 10 to study the visualization and kinesthetic capacities to perceive movement. Regarding the negative scores, for each interference (detected by the examiner or related by the participant), a negative point was given. The final questionnaire score was calculated by adding both positive scores (visualization and kinesthetic perception) and subtracting the negative scores (interferences) [26].

#### 2.5.3. Cognitive Evaluations

Due to the complexity of elaborate mental images, it was necessary that participants also made cognitive evaluations to obtain a better understanding of some aspects that may affect the capability to visualize MI.

Attentional span, planning capacity, and visual–spatial (VS) working memory were evaluated to detect if there could be any relationship between the capacity to visualize the movement mentally and its impact on the affected UL movement. The tools employed are described as follows: (1) digit span test [45] is included in the Wechsler Adult Intelligence Scale (WAIS) and the Wechsler Memory Scales (WMS). Participants read a sequence of numbers and are asked to repeat the same sequence back to the examiner in order (forward span) or in reverse order (backward span). Forward span captures attention efficiency and capacity. Backward span is an executive task particularly dependent on working memory. The digit span subtest can be scored as one summary value (this is the score that is age normed and contributes to summary scores in the Wechsler tests) or separately for forward and backward performance; (2) block design test [45] is a subtest that is administered as part of several of the Wechsler Intelligence tests, including the Wechsler Preschool and Primary Scale of Intelligence (WPPSI, the Wechsler Intelligence Scale for Children-fourth edition [46]) and the Wechsler Adult Intelligence Scale-fourth edition [45]. It is the main measure of VS and organizational processing abilities, as well as nonverbal problem-solving skills. As this is a time-based task, it is also influenced by fine motor skills. The individual is presented with identical blocks with surfaces of solid red, surfaces of solid white, and surfaces that are half red and half white. Using an increasing number of these blocks, the individual is required to replicate a pattern that the test administrator presents to them—first as a physical model and then as a two-dimensional picture; (3) zoo map test [47] is adapted and validated in the Spanish population. The test belongs to the behavioral assessment of dysexecutive syndrome (BADS). It measures executive functions, in particular the ability to organize, plan, and solve problems in order to achieve an objective. This is a test of planning. It provides information about the participant’s ability to plan a route to visit 6 of a possible 12 locations in a zoo, first in a demanding, open-ended situation where little external structure is provided and then in a situation that involves simply following a concrete, externally imposed strategy.

#### 2.5.4. Independence of Basic and Instrumental Activities of Daily Living

(1) Barthel index (BI) is adapted and validated to the Spanish population. The Barthel index is a valid measure of disability [48] that assesses the autonomy of basic activities in daily life. Additionally, it is an important method to evaluate the capacity of participants to conduct 10 different ADLs, considered as basic ADLs, making it possible to obtain a quantitative estimation about their independence level. The values assigned to each activity are based on the time and quantity of physical assistance required by the participant; (2) the Lawton instrumental activities of daily living scale (IADL) is an appropriate instrument to assess independent living skills [49], which is adapted and validated in the Spanish language. The scale covers eight functional domains: using the telephone, shopping, food preparation, housekeeping, laundry, transport, medication, and finances. Competence is rated according to descriptions of the subject’s level of involvement/ability in each activity.

## 3. Results

### 3.1. Comparison among Groups regarding Their Capacity to Visualize MI

After analyzing the obtained results, the participants were divided into two groups depending on their ability to visualize MI (Table 1): Group-1 (G1) with a poor ability to visualize and Group-2 (G2) with a better ability to visualize MI. The average value of CEMIMA test results was employed to distinguish participants. G1 had an average value of 31.73 (SD = 11.66) and G2 had an average value of 60.20 (SD = 7.17).

The differences were statistically significant (*p* ≤ 0.05) for BI, block design, ABILHAND, FMA-UE and all its subtests (arm, wrist, hand, coordination, sensation, passive movement, and pain), 9-HPT, JHFT, and dynamometry (see Table 1).

### 3.2. Correlations between Mental Imagery and Motor Function Measures

Table 2 shows the correlations between MI and motor function measures. There is a highly significant correlation between the motor function measures ABILHAND, FMA-UE, 9-HPT, JHFT, and dynamometry and the MI evaluated through CEMIMA. There is a strong correlation between the motor function measures, ABILHAND, FMA-UE, 9-HPT, JHFT, and dynamometry and the MI evaluated through CEMIMA.

### 3.3. Correlations Cognitive Function Measures

There is a statistically significant relationship between CEMIMA and block design; hence, there is a correlation between the VS function and the ability to visualize MI. Results obtained from other cognitive instruments did not depict higher correlations with the other cognitive tests—namely, digit span and zoo map tests (Table 2).

Regarding the correlations between cognitive functions and UL movement, it is important to mention there is a statistically significant correlation between the block design test and all the UL movement scales analyzed (ABILHAND, FMA-UE, 9-HPT, JHFT, and dynamometry), but no correlation is observed between digit span and zoo map tests and the UL scales, although zoo map test and 9-HPT are related (Table 2).

### 3.4. Time since Injury and Groups

As previously described, participants were divided into two groups of early post-stroke participants (up to 6 months after stroke) and late post-stroke participants (over 6 months); as such, two different profiles are expected, where participants with a longer period from injury have poorer performance on motor imagery. 

The χ^2^ was carried out across the time after injury groups and the evocation groups. No statistically significant differences were found across them (*p* > 0.05), indicating that groups were homogeneously distributed (Table 3).

After checking the underlying assumptions (Levene’s *p* = 0.07 and qqplots), the ANOVA in CEMIMA scores indicated that differences across groups were statistically significant: F_(1,35)_ = 72.95; MSE = 6020.65; *p* < 0.01; η^2^ = 0.638. As expected, differences across time groups after injury also reached the statistical level: F_(1,35)_ = 6.35; MSE = 534.70; *p* < 0.0; η^2^ = 0.05. No interactions were found across factors under study.

## 4. Discussion

MI analysis is a complex process full of difficulties related to this therapeutic method. Nevertheless, the question of how the visualization of MI can be a useful therapeutic tool is of interest for many fields. The literature seems to indicate that MI reproduces motor efference and might be limited by an individual’s normal motor experience [50] This theory postulates that the motor schema involved in real activity is reinforced during the visualization of MI. Particularly, the motor skills are reinforced when those images involve the same motor schemas as the real movement [51].

In many studies related to the visualization of MI after a stroke [9,52,53], the quality of the visualization is considered a useful element in different treatment techniques. Conversely, the objective of our research was different—we aimed to examine whether the ability to visualize MI was related to the capacity to move the UL and understand which other variables could affect the quality of the visualization.

The main findings could be described as follows: First, a statistically significant correlation between MI and motor function measures was found. This result suggests a strong relationship between the ability to visualize MI with the UL movement and functionality after a stroke, supporting previous results in which functional equivalence between motor images and motor execution is claimed [28,29,30].

The relation between MI and movement is clear—participants were not able to perform the movements if they could not visualize them; as claimed by Olsson et al., “*If you cannot do it, you will not think it*” [33]. In several CEMIMA items, the participants were challenged to visualize a ball in their hand, and subsequently, they had to throw and catch the ball repeatedly. One result of interest in this test was related to how participants with the worst MI visualization ability had a bad movement performance. Surprisingly, we observed that some participants reported that during the visualization, they had dropped the ball or they had not been able to throw it.

Second, there is a statistically significant relationship between the block design test, which measures the VS working memory and UL evaluation instruments (FMA-UE, 9-HPT, JHFT, and dynamometry). Therefore, the participants with poorer VS skills had a greater alteration in their capacity to move the UL. Their hindrance to managing the MI was linked to the neural network disorganization, making it impossible for them to perform a UL movement correctly.

Third, there is a relationship between MI and cognitive function measures, especially VS working memory and their capability to visualize MI.

As a result of the two previous statements, it would be essential to determine whether the participants’ inability to generate MI is related to an alteration in their cognitive capacity, or whether it is due to an alteration of the motor planning caused by the brain injury. Therefore, future lines of research should address whether poorer cognitive mechanisms to perform MI visualization are due to an alteration in their VS skills, or motor planning is damaged, and therefore, individuals cannot access it. Consequently, it seems important to emphasize two statistically significant correlations related to VS skills. On the one hand, VS with the MI visualization and, on the other hand, VS with the affected UL movement. These correlations allow us to conceptualize how motor and cognitive systems are strongly interrelated and, therefore, cannot be separated in neurorehabilitation.

The literature has addressed the relationship between cognition and movement [54,55,56]. An example of the importance of the movement mental representation and mental images manipulation occurs in the rehabilitation technique called graded motor imagery when training the perception of laterality. This training is based on judging, through a sequence of photographs, if the part of the body shown corresponds to the right or left half-body, where the participants have to perform an adequate mental rotation using the working memory process [57]. It is considered that these types of cognitive strategies, as well as others related to the involvement of cognitive functions in motor function, can be very useful in UL rehabilitation [58].

Lastly, two well-defined groups of participants were of interest according to their MI ability. G2 had a relatively normal ability to visualize MI, while, the other group had a greater impediment to achieving it (G1). The latter group, G1, had a lower ability to visualize MI, poorer VS, worst dexterity, less strength, and consequently a reduction in their UL mobility and functionality; additionally, they were more ADLs dependent. Future research should examine these profiles, which may set standardized criteria and approaches for the MI technique and would be very useful to the neurorehabilitation scientific community.

To conclude, it should be noted that the ability to visualize MI and cognitive functions such as VS and UL movement seem to be related. Thus, it is important to approach them all together during the treatments. This could be of interest for new rehabilitation techniques seeking to induce a cortical reorganization that would be associated with the motor relearning through the achieving and improvement in the motor function [59]. In summary, in these techniques, we can find motor imagery, action observation therapy, or mirror therapy. One should bear in mind that these therapies have a multitude of benefits: they are low cost because they do not require expensive tools such as robots; furthermore, they allow the development and use of emerging technologies such as non-immersive virtual reality (VR) applications (e.g., phones and tablets), which could be used as therapeutic supplements, particularly when physical movement is limited, enabling participants to continue their treatment in their home after finalizing the treatment session; our phones or tablets could simply be enough for allowing the subjects to continue their treatment in their home after finalizing the treatment session.

As a statistically significant relationship was found between the ability to visualize MI and the time elapsed since the stroke, it seems important to note that the longer it was since the stroke, the more likely it was to observe a lower capability to visualize MI. This could be related to the decrease in the cortical representation of the UL, probably caused by disuse, which, in turn, was generated by their incapacity to move. Likewise, time after injury is a question of interest from two aspects in applied research. On the one hand, research in this area might help to improve current theoretical models. On the other, one should bear in mind that for specific interventions to be effective, therapy must be initiated as soon as possible. Current results support the last statement, also suggesting a lack of interaction with evocation groups.

The main study limitations are related to the characteristics of the research design. It was not possible to establish control procedures for potential confounding variables to avoid certain biases in results. Future lines of research should include the results of other techniques such as neural electronic physiological exams. Moreover, and as is sometimes the case in studies with clinical samples, a small sample size was employed, which limited the generalization of results. On the other hand, the time span between the incidence of stroke and the assessment variation is a question that caused variation in the current results, as it was addressed in a dichotomous way. However, the study makes it possible to establish the possible relationship between the variables involved to conduct analytical studies, and it is expected this could be of interest for future lines of research that address these issues. Nevertheless, the results in this study seem to support previous studies in the field of MI research. Increasing the number of studies of motor imagery would shed light on the use of this technique as means of therapy.

## 5. Conclusions

We can conclude that the capacity to visualize mental images and the capacity to produce a movement in the UL after a stroke are related to each other. Additionally, we also found other correlations between VS skills and the capacity to visualize MI, as well as with the UL movement.

These results are of interest for neurorehabilitation proposes based on the improvement of MI, which could be beneficial for the UL movement and functionality rehabilitation. Including use-dependent plasticity therapies such as imagery motor, action observation therapy, or mirror therapy could increase the chances of success.

Lastly, the appearance and increased use of emerging ICT in rehabilitation bring forth new possibilities for therapists and their patients to implement activities that allow them to achieve their objectives.

## Figures and Tables

**Table 1:** Comparison among visualization of MI groups.

Evaluations	Group 1 Poor Ability to Visualize Group		Group 2 Better Ability to Visualize Group		Sig **
Variables	Average (SD *)	CI 95%	Average (SD *)	CI 95%	
Age	62.21 (7.73)	58.48–65.94	69.90 (10.41)	65.02–74.78	0.13 t
Months since injury	9.11 (10.94)	3.83–14.38	6.00 (7.26)	3.30–10.10	0.412 Z
CEMIMA	31.73 (11.66)	26.11–37.36	60.20 (7.17)	56.84–63.55	0.000 t
BI	53.16 (35.28)	36.15–70.16	82.00 (30.49)	67.73–96.27	0.005 Z
IADL	3.16 (2.98)	1.72–4.60	4.40 (2.60)	3.18–5.62	0.125 Z
ABILHAND	36.95 (59.07)	8.48–65.42	139.55 (43.71)	119.09–160.01	0.000 Z
FMA-UE	26.42 (20.55)	16.51–36.33	55.70 (15.74)	48.33–63.07	0.000 Z
FMA-UE Arm	13.84 (11.27)	8.41–19.27	30.15 (8.71)	26.07–34.23	0.000 Z
FMA-UE wrist	3.11 (3.97)	1.19–5.02	8.45 (2.92)	7.08–9.82	0.000 Z
FMA-UE hand	5.42 (5.43)	2.80–8.04	12.10 (3.89)	10.28–13.92	0.000 Z
FMA-UE sensitive	7.32 (4.04)	5.37–9.26	10.15 (2.88)	8.80–11.50	0.014 Z
FMA-UE coordination	4.00 (1.29)	3.3820134.62	4.95 (1.39)	4.30–5.60	0.023 Z
FMA-UE Pain	20.47 (4.33)	18.38–22.56	22.75 (2.59)	21.54–23.96	0.034 Z
FMA-UE Mvt. Pas	21.9 (2.29)	20.68–22.90	23.10 (1.61)	22.34–23.86	0.008 Z
JHFT	671.37 (313.63)	520.21–822.54	210.13 (236.65)	99.37–320.89	0.001 Z
9-HPT	102.74 (35.53)	85.62–119.87	61.41 (40.45)	42.47–80.34	0.005 Z
Dynamometry	4.25 (6.45)	1.14–7.36	17.32 (11.48)	11.54–22.18	0.000 t
Zoo map test	1.47 (1.32)	0.79–2.15	1.58 (1.64)	0.79–2.37	0.935 Z
Block design	7.42 (3.30)	5.83–9.01	9.55 (3.22)	8.04–11.06	0.049 t
Digit span	10.56 (3.11)	8.90–12.22	10.15 (2.34)	9.05–11.25	0.700 Z

* SD: standard deviation ** Sig: significance. Depending on the normality, the Mann–Whitney U or *t*-test was used to determine significance, with Student’s *t*-test and a z for the Mann–Whitney U. CEMIMA: the mental evoking images, movements and activities questionnaire, FMA-UE: Fugl–Meyer assessment for the upper extremity, JHFT: the Jebsen hand function test; BI: Barthel index; IADL: Lawton instrumental activities of daily living scale; 9-HPT: nine-hole peg test.

**Table 2:** Pearson’s correlation coefficient between MI and motor and cognitive function measures.

Variables	1	2	3	4	5	6	7	8	9	10	11	12	13
1.CEMIMA	--												
2.FMA-UE	0.74 **	--											
3. 9-HPT	-0.58 **	-0.84 **	--										
4.JHFT	-0.71 **	-0.94 **	0.87 **	--									
5. Dynamo	0.65 **	0.81 **	0.80 **	−0.77 **	--								
6. ABILHAND	0.77 **	0.94 **	0.80 **	−0.97 **	0.77 **	--							
7. Block D	0.54 **	0.43 **	−0.34 *	−0.41 *	0.37 *	0.43 **	--						
8. Digitos	−0.48	-0.90	0.83	0.59	−0.10	−0.11	0.36 *	--					
9. Zoo Map	0.18	0.28	−0.32 *	−0.24	0.33 *	0.18	0.20	0.32	--				
10. Age	0.25	0.23	−0.16	−0.34 *	0.12	0.34 *	−0.13	0.10	−0.13	--			
11. M.S.Inj	−0.35 *	−0.27	0.26	0.33 *	−0.25	0.34 *	−0.42 **	0.02	−0.02	−0.27	--		
12. IADL	0.34 *	0.55 **	−0.46 *	−041 *	0.45 *	0.41 **	0.16	−0.09	0.36 *	−0.35 *	0.28	--	
13. BI	0.47 **	0.62 **	−0.55 **	−0.48 **	0.56 **	0.52 **	0.17	−0.21	0.25	−0.46	0.18	0.81 **	--

Level of significance evaluations: * *p* < 0.05, ** *p* < 0.001. FMA-UE: Fugl–Meyer assessment for the upper extremity; JHFT: the Jebsen hand function test; 9-HPT: nine-hole peg test; Dynamo: dynamometry; Block D: block design; Zoo Map: zoo map test; M.S. inj: months since injury; IADL: the Lawton instrumental activities of daily living scale; BI: Barthel index.

**Table 3:** Contingency tables across evocation groups and early and late post-stroke period.

Participants		Time After Injury		Total
Group	Counts	< 6m	> 6m	
Lower	Count	10	9	19
	% within row	52.632 %	47.368 %	100 %
	% within column	40.000 %	64.286 %	48.718 %
	% of total	25.641 %	23.077 %	48.718 %
Higher	Count	15	5	20
	% within row	75 %	25 %	100 %
	% within column	60 %	35.714 %	51.282 %
	% of total	38.462 %	12.821 %	51.282 %
Total	Count	25	14	39
	% within row	64.103 %	35.897 %	100 %
	% within column	100 %	100 %	100 %
	% of total	64.103 %	35.897 %	100%

## Data Availability

The data that support the findings of this study are available from the corresponding author on reasonable request.
